# A novel and efficient approach to high-throughput production of HLA-E/peptide monomer for T-cell epitope screening

**DOI:** 10.1038/s41598-021-96560-9

**Published:** 2021-08-26

**Authors:** Juliette Vaurs, Gaël Douchin, Klara Echasserieau, Romain Oger, Nicolas Jouand, Agnès Fortun, Leslie Hesnard, Mikaël Croyal, Frédéric Pecorari, Nadine Gervois, Karine Bernardeau

**Affiliations:** 1 P2R “Production de Protéines Recombinantes”, Université de Nantes, CRCINA, SFR-Santé, INSERM, CNRS, CHU Nantes, Nantes, France; 2 Université de Nantes, Inserm, CRCINA, 44000 Nantes, France; 3 LabEx IGO «Immunotherapy, Graft, Oncology», Nantes, France; 4 Université de Nantes, CHU Nantes, Inserm, CNRS, SFR Santé, Inserm UMS 016, CNRS UMS 3556, 44000 Nantes, France; 5 Université de Nantes, CHU de Nantes, Cibles et médicaments des infections et du cancer, IICiMed, EA 1155, 44000 Nantes, France; 6 Université de Nantes, CHU Nantes, CNRS, INSERM, l’institut du thorax, 44000 Nantes, France; 7 CRNH-Ouest Mass Spectrometry Core Facility, 44000 Nantes, France

**Keywords:** Biological techniques, Biotechnology, Immunology

## Abstract

Over the past two decades, there has been a great interest in the study of HLA-E-restricted αβ T cells during bacterial and viral infections, including recently SARS-CoV-2 infection. Phenotyping of these specific HLA-E-restricted T cells requires new tools such as tetramers for rapid cell staining or sorting, as well as for the identification of new peptides capable to bind to the HLA-E pocket. To this aim, we have developed an optimal photosensitive peptide to generate stable HLA-E/pUV complexes allowing high-throughput production of new HLA-E/peptide complexes by peptide exchange. We characterized the UV exchange by ELISA and improved the peptide exchange readout using size exclusion chromatography. This novel approach for complex quantification is indeed very important to perform tetramerization of MHC/peptide complexes with the high quality required for detection of specific T cells. Our approach allows the rapid screening of peptides capable of binding to the non-classical human HLA-E allele, paving the way for the development of new therapeutic approaches based on the detection of HLA-E-restricted T cells.

## Introduction

Human leukocyte antigen E (HLA-E) is a highly conserved non-classical class Ib MHC molecule that is currently attracting renewed interest among immunologists due to its highly conserved nature and its rather unique dual properties in both innate and adaptive immunity. Although 43 HLA-E alleles have been reported to date (IPD-IMGT/HLA Database^1^), most of them are either very rare or encode only non-functional proteins. In fact, two main allelic forms together account almost for 100% in human population. These two alleles, HLA-E*01:01 and HLA-E*01:03, differ by a single amino acid substitution (Arg or Gly) at position 107, located on a loop outside the peptide binding groove^2,3^. They are distributes worldwide in roughly equal proportions and have a relatively low basal expression at the cell surface compared to that of HLA class Ia (classical), as HLA-A and HLA-B molecules^4^.

Under normal circumstances, HLA-E exhibits preferential binding to a highly conserved set of nonameric signal peptides (VL9) derived from the leader sequence of HLA class Ia molecules and HLA-G^3^. The recognition of these complexes by the heterodimeric CD94/NKG2 receptor family represents a central innate mechanism by which NK cells indirectly monitor the expression of other HLA molecules in physiological and pathological conditions^5,6^. However, HLA-E could also be involved in the adaptive surveillance of non-self, as it also displays features of an antigen-presenting molecule for CD8 αβ T cells. In pathological and/or stress situations, HLA-E is associated with the replacement of bound signal peptides by a novel much more diverse repertoire of peptides, which can be sensed by αβ T-cell receptors (TCRs)^7^. Thus, over the last two decades, an increasing number of studies have reported the existence of HLA-E-restricted T cells during various bacterial and viral infections, highlighting their contribution to host defense. As examples, cytolytic CD8 αβ T cells recognized HLA-E presented peptides derived from persistent infections with bacteria, such as *Salmonella enterica serovar Typhi*^8^, *Listeria monocytogenes* and *Mycobacterium tuberculosis*^9–11^ as well as viruses, such as hepatitis C virus^12^, Epstein-Barr virus (EBV)^13^ and human cytomegalovirus (HCMV)^14–17^. These pathogen-specific HLA-E-restricted CD8^+^ T cells are an interesting new player in the immune response, which has also been recently highlighted in the response to SARS-Cov-2^18^, and could therefore be targeted for the development of new immunotherapies. The diversity and quality of these HLA-E-restricted T-cell responses need to be further investigated. Thus, it is important to develop an approach allowing the rapid generation of HLA-E/peptide complexes for the screening of epitopes potentially recognized by these non-classical T lymphocytes.

MHC/peptide complexes (pMHC) have low affinity for TCR and the pMHC-TCR complex is not stable enough for an efficient labeling technique^19,20^. Therefore, pMHC must be multimerized to increase binding stability via an avidity effect. The use of tetrameric pMHC labeled with streptavidin conjugated to a fluorophore has become an essential tool for phenotyping, characterizing and isolating antigen-specific CD8 T cells^21,22^. The production of pMHC is a quite laborious and time-consuming process. Using the conventional batch production protocol, approximately 10 days are required to obtain a validated pMHC tetramer for one peptide of interest^23,24^. This production method is thus not suitable for a screening approach aimed at either designing novel epitopes for a specific MHC allele or producing tetramers for visualization of the antigen-specific T-cell population. An efficient approach for high-throughput production of pMHC complexes is therefore crucial for these purposes^25^. Several technical approaches have emerged over the past decade for classical human or murine pMHC class Ia. These are based on the use of the recombinant MHC I heavy chain properly oxidized and biotinylated in vivo^26^, a di-peptide as a chaperone for pMHC formation^27,28^, high temperature to destabilize pMHC and create another one^29,30^, or UV-sensitive peptides to produce photocleavable complexes^31–33^. These approaches coupled with a combinatorial coding strategy by flow cytometry^34^ or more recently by mass spectrometry^35^ offer a broad perspective to decipher the immune response and develop novel therapeutic strategies. In order to investigate the diversity of non-classical HLA-E-restricted T-cell responses in different pathological contexts, we developed a strategy to produce HLA-E/peptide complexes in high throughput. We defined an optimal photosensitive peptide (pUV) using UL40 signal peptides from HCMV to generate hundreds of stable HLA-E/peptide complexes within one hour. A key point for generating high quality tetramers is to accurately estimate the concentration of newly formed pMHC after UV exposure in the presence of a peptide of interest. This allows the use of the necessary amount of streptavidin to tetramerize the complexes in an optimal manner. We therefore compared two quantification methods: the widely used ELISA method and a size exclusion chromatography method that we specifically developed for peptide exchange. Finally, for functional validation of the generated MHC/peptide complexes, we compared the labeling of HCMV-specific HLA-E-restricted CD8 T cells, which we generated^14^ using either conventional batch productions or peptide exchange productions.

## Results

### Design of photocleavable peptides

**Figure 1:**
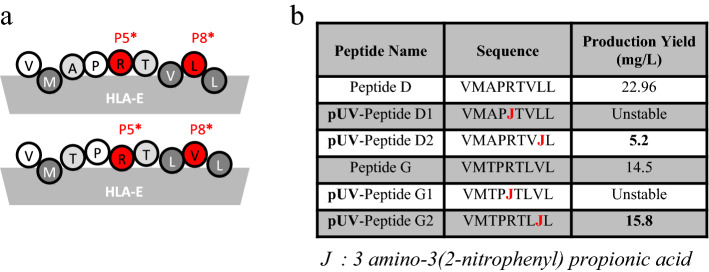
HLA-E anchor residues, UL40 peptides mutation, sequence of photosensitive peptides and production yields obtain using the batch protocol. (**a**) Schematic representation of UL40 peptides binding in the heavy chain HLA-E groove. Circles represent the peptide amino acids of the peptides. The dark ones are the majors anchor residues, the light gray ones are the minor anchor residues and the white ones correspond to those with low involvement in the binding to the HLA-E groove. The red residues (P5 and P8) are amino acid selected for UV sensitive amino acid replacement with 3 amino-3 (2-nitrophenyl)propionic acid. (**b**) Sequences of the photosensitive mutated UL40 peptides and yield obtained in batch production for corresponding HLA-E/peptide complexes.

To define photocleavable peptides, we selected 13 peptides derived from the leader sequence of different HLA class I molecules and/or HCMV UL40 (Table 1)^14,36,37^. These peptides were tested for their ability to form HLA-E/peptide monomer complexes using a batch protocol and the yield obtained with each peptide was estimated (Table 1). Among the 13 peptides tested, 11 allowed refolding of the corresponding HLA-E/peptide monomer. The other two peptides (H and L) did not allow production of stable MHC/peptide complexes despite a quite high prediction score on NetMHCpan 4.1 server compared to the others, in particular for peptide H with a score of 237.59 nM^38^. In order to design photosensitive peptides with binding and stabilization properties required for the purification process, we choose peptide D corresponding to the best production yield and peptide G corresponding to an intermediate one. For peptide exchange to occur, the complexes must be destabilized by the release of the peptide from the groove after its UV-induced cleavage. Thus, the most affine peptide may not be the best candidate. The anchor residues for peptide-binding in the HLA-E peptide groove are so far well described^39,40^. The amino acid residues at positions P2, P7 and P9 are highly conserved and correspond to major anchors for peptides into HLA-I pocket^41–43^, whereas positions P3 and P6 correspond to minor anchor residues. We therefore chose to modify the D and G peptides for TCR-directed position P5 or for the rather neutral P8 position, with the UV sensitive amino acid 3-amino-3-(2-nitrophenyl)propionic acid (J residue) to limit the impact of the mutations on the binding capability of the peptide in the MHC heavy chain groove (Fig. 1). Surprisingly, mutation at the P5 position did not allow the purification of stable pMHC with either pUV-D1 or with pUV-G1 (Fig. 1). In contrast, peptides with UV-sensitive residue at position P8 were compatible with batch pMHC productions. Indeed, we achieved production yields of 5.2 mg/L for the HLA-E/peptide D2 complex and 15.8 mg/L for the HLA-E/peptide G2. These two peptides were therefore selected for further characterization.

**Table 1:** Sequences of HLA-E/UL40 peptides and yields of corresponding complexes produced using a batch protocol.

Peptide name	Signal peptide	Origin		Production yield (mg/L)	NetMHCpan4.1 prediction score (nM)
		HLA	UL40 HCMV		
Peptide A	VMAPRALLL	HLA-C*06:17, *07, *18	+	19.6	83
Peptide B	VTAPRTVLL	HLA-B*5, *07:65, *15, *21, *35, *40 (80.3%), *41, *44:18, *45, *46, *49, *50, *51, *52, *53, *57, *58, *60, *61, *78	+	15.1	1440
Peptide C	VTAPRTLLL	HLA-B*13, *18, *27, *35:42, *37, *40 (19.6%), *44, *47, *54, *55, *56, *59, *60, *61, *82, *83	+	11.4	1027
**Peptide D**	**VMAPRTVLL**	HLA-B*07, *08, *14, *15, *38, *39, *42, *48, *62, *63, *64, *65, *67,*73, *75, *81	+	**22.9**	**88**
Peptide E	VMAPRTLIL	HLA-C*01, *03, *04, *05, *06, *08, *12, *14, *15:43, *16, *17:02	+	17.7	87
Peptide F	VMAPRTLLL	HLA-A*01, *03, *11,*29,*30,*31,*32,*33,*36, *74 HLA-C*02, *15	+	13.5	52
		HLA-C*02, *15			
**Peptide G**	**VMTPRTLVL**	-	**+**	**14.5**	**186**
Peptide H	VMAPRILIL	-	+	0	237
Peptide I	VMAPRSLIL	-	+	12.8	96
Peptide J	VMAPRSLLL	-	+	10.1	54
Peptide K	VMAPQSLLL	-	+	11.11	99
Peptide L	VMAPRTLFV	-	+	0	1062
Peptide M	VMAPRTLVL	HLA-A*02, *10, *23, *24,*25,*26, *28, *34, *43, *66, *68, *69	+	9.8	72

Peptides D and G in bold character represent peptides selected for the design of photosensitive peptides.

### Characterization of pUV peptides and HLA-E-pUV complexes

**Figure 2:**
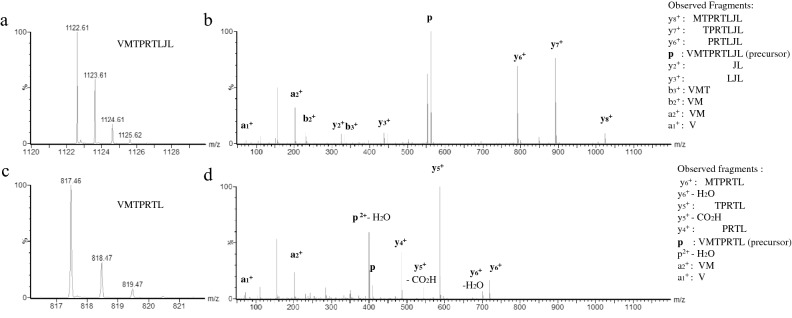
Characterization of pUV G2 peptide cleavage by mass spectrometry. Peptides were diluted to a concentration of 64.8 µM and injected onto a C18 column. Mass spectrometry profiles are shown for native pUV G2 peptide (**a**,**b**) and the cleaved G2 peptide (**c**,**d**). (**a**,**c**) represent the LC profiles with the peptide sequence. (**b**,**d**) show the MS/MS profiles with the identified fragments allowing sequence deduction.

We first tested the UV sensitivity of selected pUV peptides by liquid chromatography high resolution mass spectrometry (LC-HRMS). The pUV-D2 and G2 peptides, with a theoretical mass of 1078 Daltons and 1122 Daltons, respectively, were analyzed before and after UV-exposure (Fig. 2 and supplementary data S1). The loss of 305.15 Daltons after UV cleavage of pUV-G2 corresponds to the removal of the two last “JL” residues in the peptide sequence. These results are consistent with cleavage of the peptide exactly on the photosensitive amino acid J leading with the expected mass of 817 Da (1122- 210 (J)  − 113 (L) + 18 (H_2_O)) (Fig. 2A). Similarly, we observed a 773 Dalton peptide after UV exposure of pUV-D2 peptide ((1078 -210 (J)  − 113(L) + 18 (H_2_O)) (Supplementary data S1). These results were also confirmed by the MS/MS fragmentation patterns on the doubly-charged precursor peptide ions. We detected 8 specific ion fragments for the pUV-G2 peptide and 7 after UV cleavage (Fig. 2B), in agreement with their expected UV cleavage site given the exact peptide sequences. Consistent cleavage and sequencing were also obtained for the pUV-D2 peptide (supplementary data S1).

**Figure 3:**
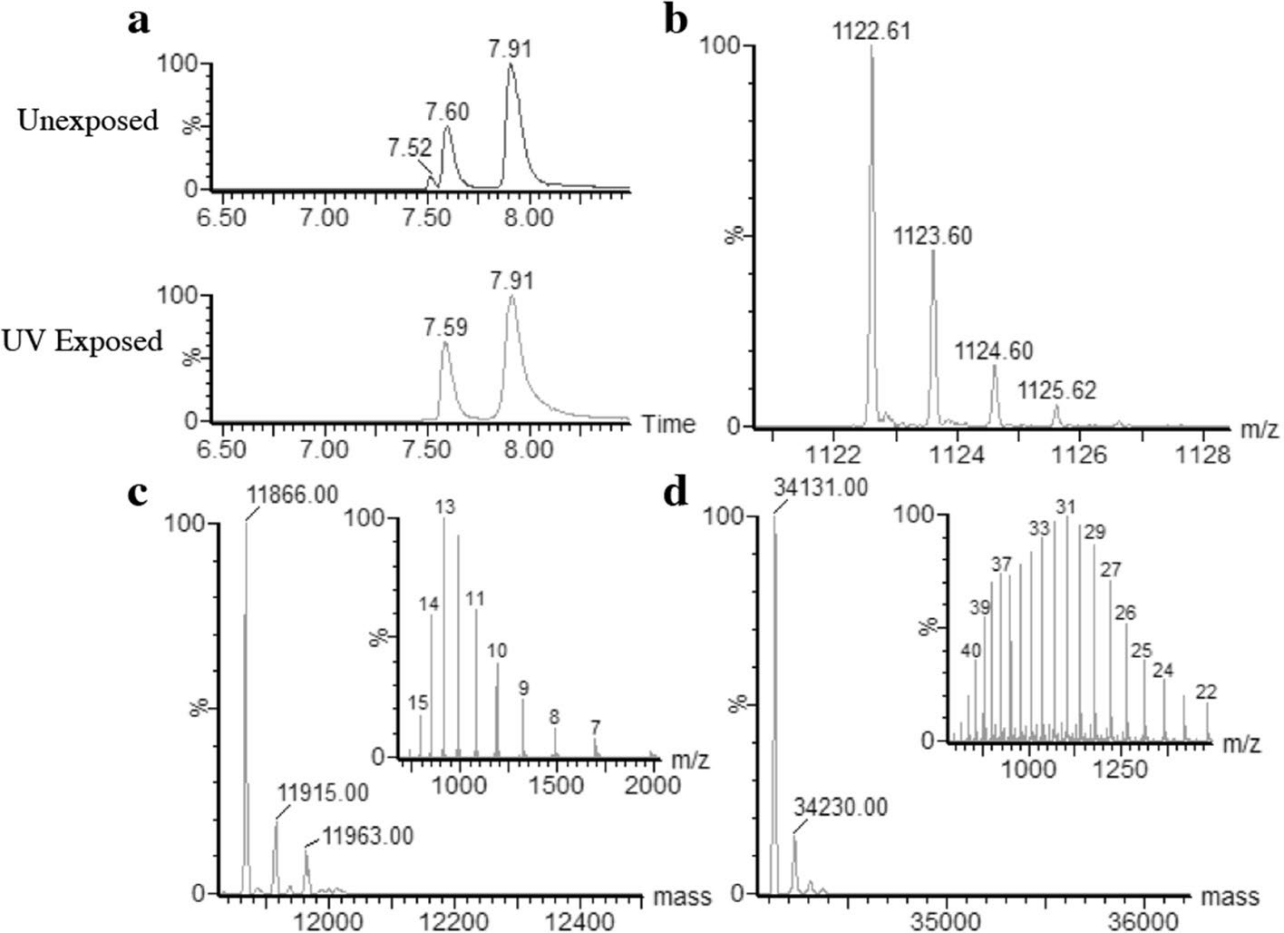
Characterization of HLA-E/pUV peptide G2 complexes by mass spectrometry. The photosensitive monomer HLA-E/pUV peptide G2 was injected onto a C18 column to analyze its components. (**a**) LC profiles of the monomer before and after UV exposure. (**b**) Mass spectrum analysis of the G2 peptide eluted in the 7.52 min peak of the unexposed monomer LC profile. (**c**) (**d**) Deconvoluted mass spectra obtained from the analysis of the chromatographic peak at 7.6 min (**c**) and 7.9 min (**d**) with mass spectra in insert.

These pUV-D2 and G2 photo-labile peptides were used to produce HLA-E/peptide complexes on a larger scale by a classical batch protocol (described in the Methods section). After biotinylation and purification, the complexes formed upon refolding of HLA-E and ß2-microglobulin chains with peptides were analyzed by LC-HRMS spectrometry. The HLA-E/pUV complexes were injected onto a UPLC column before HRMS detection to separate the heavy chain, light chain and peptide (Fig. 3A and supplementary S2 A). Without UV exposure, the pUV-G2 peptide was eluted first in the peak at 7.52 min of the elution gradient. The mass spectrum showed the expected single-charged peptide at 1122 Daltons (Fig. 3B). Deconvolution of MS spectra at 7.60 and 7.91 min of the elution gradient, allowed characterization of ß2-microglobulin light chain at 11865 Daltons (Fig. 3C) and of the heavy chain at 34136 Daltons mass, respectively (Fig. 3D). This approach allowed us to distinguish the 3 different species involved in the tested HLA-E/peptide complexes. After UV exposure, the light and heavy chains were still detected on LC profile by contrast to the pUV-G2 peptide. The same results were obtained for the HLA-E/pUV-D2 complex (Supplementary data S2).

**Figure 4:**
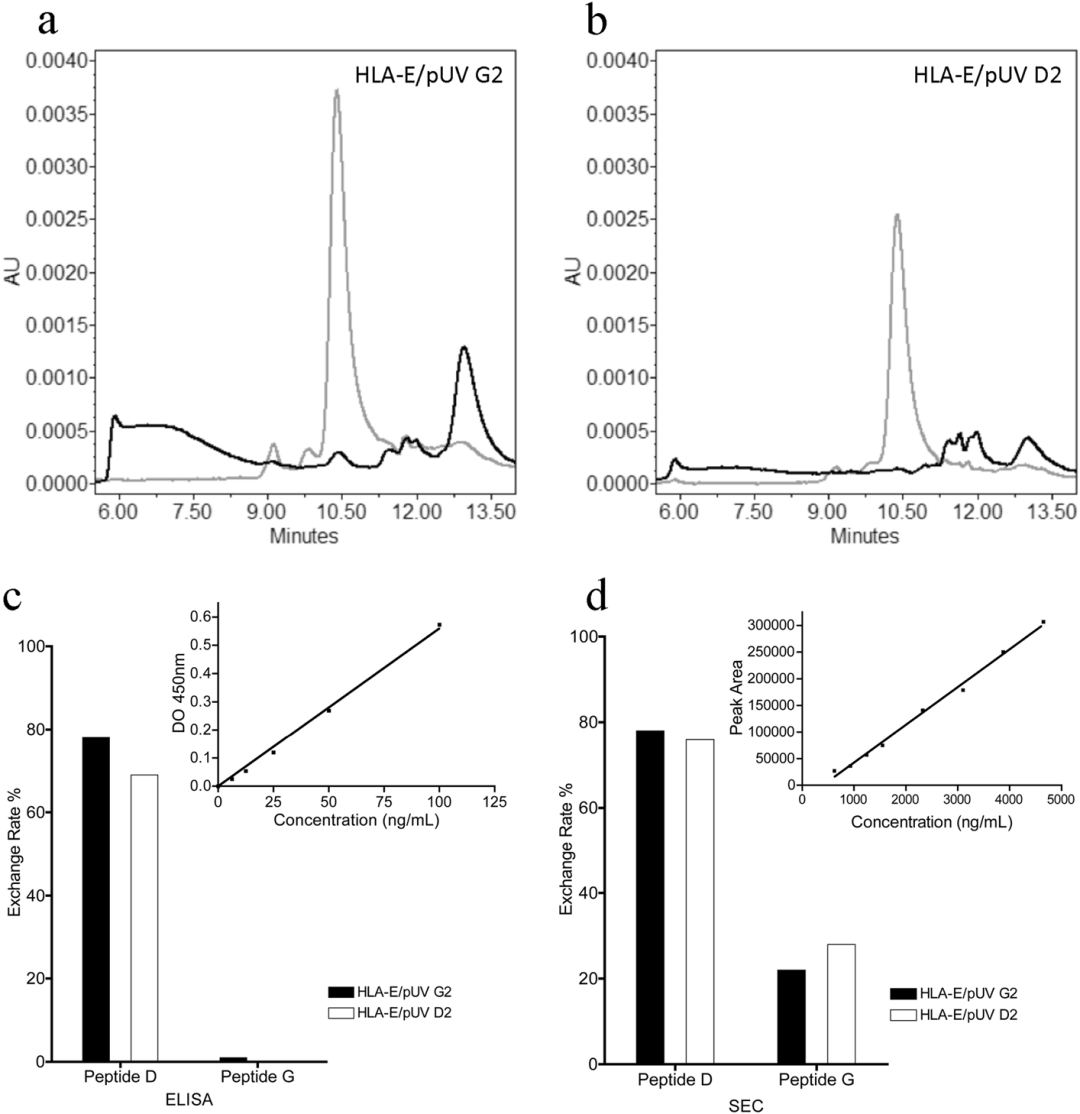
Gel filtration HPLC profiles of HLA-E/pUV complexes and comparison of neo-complex formation by ELISA and SEC quantification. Monomers were injected onto an X-Bridge BEH200Å size exclusion column and detected by absorbance at 280 nm. (**a**) Profiles obtained for the photosensitive HLA-E/pUV D2 monomer without (grey curve) or after UV exposure without rescue peptide (black curve). (**b**) Same results with the photosensitive HLA-E/pUV G2 monomer. The photosensitive HLA-E/pUV D2 and HLA-E/pUV G2 monomers were tested for their ability to form complexes with other peptides either by ELISA test (**c**) or by SEC analysis (**d**). The photosensitive monomers were exposed for 1 h at 355 nm in the presence of the native D peptide or G peptide. Exchange rates were calculated from calibration curves obtained by injecting increasing concentrations of HLA-E/Peptide D monomer produced in batch protocol (see inset for each approach).

The stability of the complexes were then tested by size exclusion chromatography before and after UV exposure. For both HLA-E/pUV-G2 (Fig. 4A) and HLA-E/pUV-D2 (Fig. 4B) monomers, a single peak was observed at 10.5 min before UV exposure, corresponding to the stable complex with a molecular weight of 48 kDa. After UV exposure, this peak disappeared to give way to aggregates and different peaks corresponding to lower molecular weights confirming the destabilization of the complex once the peptide was cleaved. The HLA-E/pUV-G2 complex was almost completely disassembled as we observed only 2.4% of intact complex persisting and no persistent HLA-E/pUV-D2 complex was observed after UV cleavage.

### Production of new HLA-E/peptide complexes

Next, we investigated whether the two UV-sensitive complexes (HLA-E/pUV D2 and HLA-E/pUV G2) could allow the production of new complexes after UV exposure in the presence of peptides of interest. We first validated the new productions with the two initial native peptides, D and G. The results were first evaluated by the conventional method, involving a specific sandwich ELISA, adapted from previous studies related to other human HLA alleles^31^. We observed a strong correlation between the concentration of a batch-produced HLA-E/D peptide complex and the optical density from which we were able to deduce a standard curve to estimate the sample concentration (Fig. 4C). The amount of newly formed complex after UV exposure, was expressed as a percentage of the initial amount of UV-sensitive complex after subtraction of any remaining uncleaved photosensitive monomer. The use of peptide D led to the production of large amounts of HLA-E/peptide complexes from both UV-sensitive complexes. Unexpectedly, we could not detect HLA-E/peptide G complex neither from HLA-E/pUV D2 nor from HLA-E/pUV G2 complexes. This result was not in agreement with the batch production of this complex, suggesting a high affinity of G peptide for HLA-E (Table 1). To better understand this discrepancy, we tested an alternative approach to detect HLA-E/peptide complex formation using size exclusion chromatography (SEC) and analytic HPLC. The insert in Fig. 4D showed the quantitative data and calibration curve obtained with batch produced HLA-E/peptide D. With this SEC analysis, we were able to detect both HLA-E/peptide D and HLA-E/peptide G complexes from the two pUV complexes demonstrating the binding of G peptide in the groove of HLA-E in accordance with other results obtained with this peptide. The quantity of complex obtained for peptide G was lower than for peptide D suggesting that peptide D has a greater affinity and/or ability to stabilize the HLA-E molecule than peptide G. Both photosensitive monomers appear to have equal overall efficiency for a given peptide. However, since the average production yield obtained for pUV complexes in batch protocol was almost three times higher for HLA-E/pUV G2 (Table 1), we chose this monomer for further experiments. Regarding the two analytical methods used, our results show that SEC analysis is much efficient than the classical ELISA approach, allowing the detection of peptides with lower affinity peptide, such as the G peptide.

**Figure 5:**
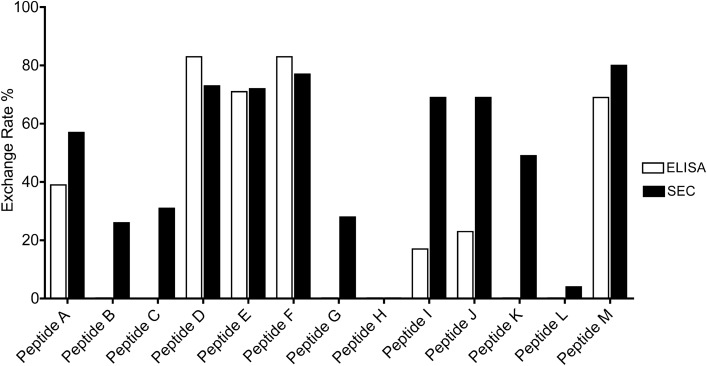
Peptide exchange screening. Comparison of the two quantification methods, ELISA and SEC. The HLA-E/pUV G2 photosensitive monomer was exposed for 1 h at 355 nm in the presence of 13 different peptides. Exchange rate was calculated as described in Methods section.

To further test the efficacy of our SEC analysis approach, we screened 13 peptides derived from the viral protein UL40^14,37^ for their ability to produce HLA-E/peptide complexes from the pUV-G2 monomer. The results obtained by classical ELISA and by SEC analysis were compared in Fig. 5. Interestingly, we observed a large discrepancy between the relative affinities of the peptides revealed by the two methods. For ELISA, 4 peptides showed high exchange rate (exchange rate greater than 50%), 3 had low ability to stabilize HLA-E/peptide complex and 6 did not show any capacity to stabilize monomers. For SEC analysis, 8 peptides showed high stabilization potential (superior to 50%), 3 showed low affinity and 2 showed no affinity. In addition, the only two peptides, H and L, that were not able to stabilize the monomer were also the only ones that failed to produce a pure stable monomer with the batch protocol (Table 1). All the other peptides allow us to produce stable complexes with a batch protocol. These results validate the SEC screening test to determine which HLA-E/peptide complexes can be produced. Indeed, SEC analysis allowed us to select 4 additional peptides with high affinity compared to ELISA (peptides A, I, J and K), highlighting the advantage of this technique over ELISA.

### Functional validation of HLA-E/peptide complexes: detection of antigen-specific T-cell responses with HLA-E exchange tetramers

**Figure 6:**
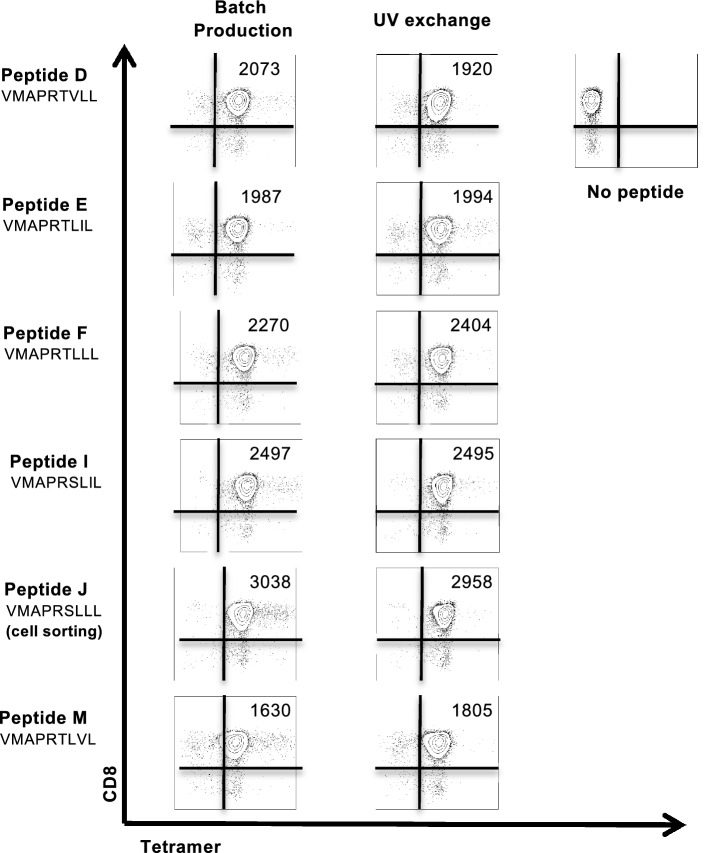
Detection of antigen-specific T-cell responses with HLA-E exchange tetramers. HCMV_UL40_-specific CD8 T cells were stained with equal amounts of six HLA-E UL40 tetramers generated by classical batch refolding (left) or peptide exchange (right). Numbers indicate the mean fluorescence intensity (MFI) of tetramer-positive cells.

We first checked the production efficiency of the complexes generated by peptide exchange under conditions compatible with the screening of many peptides, i.e. in a very short time (1 h) and in a microplate format. In order to validate the functionality of the complexes produced, we tested their recognition by T cells, their natural target. For this purpose, we used HLA-E-restricted CD8 αβ T cells specific for HCMV UL40 signal peptides, which we magnetic-sorted from PBMCs of a kidney transplant patient^14^. We produced monomers refolded with 6 different UL40_15-23_ peptides by conventional or peptide-exchange technology. After estimating their concentrations, these monomers were tetramerized with phycoerythrin-coupled streptavidin to label specific CD8 T cells. We then compared the staining of HCMV-committed HLA-E-restricted CD8 αβ T cells using both types of tetramers. As shown in Fig. 6, the results obtained with the two approaches were similar both in terms of the percentage of positive HCMV-specific HLA-E-restricted CD8 T cells (more than 95% in all cases) and of the mean fluorescence intensity of these cells (MFI). As expected, the most significant response (MFI of 3038 and 2958 with HLA-E classical and exchange tetramers, respectively) was obtained with the HLA-E tetramer loaded with the UL40_15-23_ peptide VMAPRSLLL (peptide J) corresponding to the patient’s own HCMV infecting strain and which was used for HCMV-specific cell sorting. Moreover, and as described previously^14^, consistent staining was also obtained for HLA-E tetramers loaded with the other five peptides. Nevertheless, as shown by the results obtained with peptide M (VMAPRTLVL), changing in P8 or P6 residues of UL40_15-23_ peptides led to a lower intensity labeling (MFI of 1630 and 1805 with HLA-E classical and exchange tetramers, respectively) reflecting the relative importance of these two amino acids for the interaction of HLA-E complexes with the TCR. Thus, this approach allowed rapid generation of HLA-E/peptide complexes while maintaining their functional conformation and activity.

## Discussion

In order to investigate antigen specific CD8 T-cell response, MHC/peptide tetramers have become a key tool. A major drawback of the high throughput use of multimer form of MHC/peptide complex is that for each specific complex, separate production is required^23^. To overcome this problem, many approaches have been reported among them the use of photocleavable peptides described for classical human or murine MHC-I alleles^20,31,44^. In this approach, a unique and stable MHC/pUV complex produced in a bath and stored at − 80 °C, is used to be exchanged with peptides of interest after UV exposure in microplate format.

Here, we present a rapid and powerful approach combining the use of novel photocleavable peptides and accurate monitoring of the exchange by SEC analysis to allow efficient productions of HLA-E/peptide monomers. We first characterized the best photocleavable peptides capable of stabilizing HLA-E complexes and being completely cleaved after 1 h of UV exposure. The peptide binding groove of HLA-E accommodates 9-mer peptides through the major anchor residues at positions P2, P7 and P9^39^. The amino acids at position P5 and P8, on the other hand, are more critical for the interaction of the HLA-E/peptide complex with the CD94/NKG2A/C receptor and with the TCR of specific T cells^41,45^. We chose to modify two UL40_15-23_ peptides, VMAP**R**TV**L**L (D peptide) and VMTP**R**TL**V**L (G peptide), with high affinity for HLA-E and which allowed us to achieve high yields in classical monomers production. We mutated the P5 or P8 positions of the D and G peptides with the photosensitive residue J almost described in the literature^31,32^. Using the mutated P8, D2 (VMAPRTV**J**L) or G2 (VMTPRTL**J**L) peptides, we obtained a high yield of stable HLA-E/peptide complexes almost fully cleaved by UV exposure. Mass spectrometry confirmed UV cleavage of the **J**L fragment and photocleavable complexes were formally validated by LC-HRMS and MS/MS analyses. This mutation did not influence the yield of complex production for peptide G but reduced the yield for peptide D by a factor of 4 (Fig. 1). Substitution of hydrophobic leucine with a photosensitive residue appears to impact peptide binding in the groove of HLA-E even though it allowed stable purification of the complex. The mutation of the arginine residue at position P5 by residue J in peptides D and G did not allow us to purify sufficient stable complexes. This position is known to be solvent oriented and we expected that its mutation would not significantly interfere with peptide binding in the HLA-E pocket groove. However, our results showed that modification of the residue at this position induced a strong decrease in affinity and/or stabilization ability to form HLA-E/peptide monomers. Recent studies on the peptidome related to human HLA-E and primate or murine HLA-E homologues^42,43^ have assessed the plasticity of the HLA-E pocket using the photosensitive peptide VMAP**J**TLVL. This UV peptide, mutated at the P5 position differs only at the P7 or P3 position from our D1 and G1 peptides (VMAP**J**TVLL and VMTPJTLVL respectively), both of which were inefficient in this study. The discrepancy observed for the D1 peptide could be due to the presence of hydrogen bond between the peptide backbone at P7 and the peptide binding groove of HLA-E, and the interaction between the adjacent P6-P7 side chains in the hydrophobic pocket described by O’Callagan^41^. As with the G1 peptide, while the P7-P9 LVL sequence allows good binding to the HLA-E heavy chain pocket (VMAP**J**TLVL^43^), the modification of an alanine with a threonine at position 3 could prevent peptide binding to the groove.

Once HLA-E/peptide complexes are formed after peptide exchange, their quantification is crucial for the subsequent production of tetramers. Indeed, an under- or over-estimation of the concentration of newly formed monomers would lead to the formation of a mixture of mono-, di-, tri- and tetramers with a consequent decrease of T cell labeled by these complexes and thus a bias in this analysis. Therefore, it is crucial to develop a robust method for monomer quantification. We first used the widely used ELISA approach and adapted the previously described sandwich ELISA for other HLA class I allele^31^ to HLA-E/peptide detection. Biotinylation of the heavy chain of HLA-E/pUV monomers allowed their capture by streptavidin coated on the bottom of ELISA wells. Detection of the immobilized monomer was performed using an anti-ß2m-HRP antibody. This specific ELISA has the advantage of detecting specifically assembled heavy and light chains. This approach is very sensitive, allowing the detection of very low concentrations of monomer in the range of 10 to 100 ng/mL. However, we observed that some HLA-E/peptide monomers were not detected by ELISA although they were clearly formed as shown by other tests (good quality batch production and purification, positive specific T-cell labeling). We successfully detected these monomers by SEC with a peak corresponding to the expected retention volume (Fig. 4). The SEC calibration curve allowed reproducible sample quantification and was suitable for the low peptide exchange volume. Automated injections using the Alliance HPLC system (120 vials) coupled with the automatic analysis method allow the rapid quantification of monomers without human intervention after column preparation and sample transfer to the vials.

The discrepancy observed for some peptides between the two readout methods probably results from the different dilution factors of the exchange reactions. Indeed, peptide exchange is more efficient at high concentrations of pUV complexes (data not shown), leading to the need of a very high dilution factor of the exchange reaction (at least 1/5000) before quantification by ELISA. Conversely, the chromatography method, which remains less sensitive than ELISA (standard curve quantification above 100 ng/mL) required a lower dilution (usually 1/5). The binding of the peptide to the heavy chain groove is a balance between association and dissociation of the peptide (k_on_ and k_off_). High dilution of the sample, and thus of the peptide concentration in the solution, could shift this equilibrium towards dissociation of the HLA/peptide complex, ultimately leading to dissociation of HLA/ß2m because this heterodimer is unstable without peptide bound to the HLA groove. This observation is in line with the fact that (i) peptides with low binding affinity exhibit a decrease in thermal stability (Tm value) conferring instability of the complex^44^ and (ii) the different behaviors of low and high affinity peptides for their denaturation process^27,46^. Furthermore, the fact that HLA-E molecules are less stable than other class I alleles led us to adapt the complex purification process with milder purification steps (data not shown). Taken together, these observations suggest that this relative instability makes HLA-E less compatible with the ELISA approach than more stable HLAs, such as HLA-A2 or HLA-B7, hence the need to use the SEC analysis that we have developed.

The applications of our approach will be essential in the study of non-classical HLA-E-restricted CD8 T cells. Indeed, this population has recently been considered as an interesting new player in the field of immune response^47^. Thus, the presentation of non-canonical peptides by HLA-E, especially in the absence of HLA class I molecules, is observed in various pathological conditions leading to the stimulation of these unconventional cells. Specifically, HLA-E-restricted CTLs recognizing pathogen-derived peptides have been documented in bacterial (*Mycobacterium tuberculosis* and *Salmonella enterica*)^9–11,48,49^ and viral infections (HCMV, EBV^14,15,17^) revealing the likely role of these cells in host defense, complementary to that of conventional HLA-Ia-restricted CTLs^8,50–52^. In some HCMV infections, HLA-E-restricted T cells may even play a major role in anti-virus defenses, given the over-expression of HLA-E molecules and down-regulation of HLA-I molecules. Interestingly, in a macaque model, CMV-vectored vaccines against simian immunodeficiency virus (SIV) have been shown to prime Mamu-E-restricted CD8^+^ T cells which the recognize peptide targets on SIV infected cells after viral challenge^53^. In human, Hannoun et al. recently identified some HIV-1-derived peptides that can potentially bind to HLA-E^54^ and the use of HLA-E-restricted CD8 T cell to improve immunotherapy in COVID-19 patients has recently been proposed^18^. In cancer, no tumor-derived peptides with binding specificity for HLA-E have been identified to date. However, the potential role of HLA-E as antigen-presenting molecule for tumor-specific CD8^+^ T cells should be investigated because of the frequent overexpression of HLA-E in some cancers such as melanoma, colorectal carcinoma and cervical adenocarcinoma^55–58^. All these results converge towards an emerging role for HLA-E-restricted CD8^+^ T lymphocytes in the adaptive immune response to pathogens and tumors^59^. Furthermore, because of the limited polymorphism of HLA-E, these data open up therapeutic perspectives in various pathologies in all individuals, regardless of HLA-I genotype. Moreover, Oliveira et al*.*^60^ identified peptide ligands of the mouse HLA-E homologue Qa-1b, in a TAP-deficient mouse cell line, which triggered a strong cytotoxic T-cell response. Clearly, there is an ongoing need to analyze and understand HLA-E restricted peptides in cells that are affected by tumor- or virus-induced alteration of the HLA class I presentation machinery.

In conclusion, the novel and robust approach described here is of great interest to decipher epitopes recognized by HLA-E-restricted CD8 T cells as it allows the identification of relevant peptides not detected with conventional ELISA readout. We believe that this approach will be useful for two major applications: epitope screening and detection of specific T lymphocytes by tetramer stimulation and labeling in different pathologies (bacterial, viral, tumor). In the case of cancer, for example, the precise identification of neoepitopes derived from DNA mutations and recognized by specific HLA-E-restricted T lymphocytes would allow their use as targets in the development of new immunotherapies^61^. Next-generation sequencing provides information on mutational status and allows the design of putative HLA-E binders using peptide binding algorithms developed for many HLA alleles, including HLA-E (NetMHCPan4.0^62^). This affinity score prediction will be complementary to our proposed binding test, as discordance between the results obtained by these two assays has been observed for some peptides (Table 1)^44^.Finally, our SEC-based quantification approach allows the generation of HLA-E/peptide tetramers that are effective for phenotyping, characterizing, and sorting restricted HLA-E CD8 T-cell populations and could improve immunotherapies approaches.

## Methods

### Peptides

The peptides A (VMAPRALLL), B (VTAPRTVLL), C (VTAPRTLLL), D (VMAPRTVLL), E (VMAPRTLIL), F (VMAPRTLLL), G (VMTPRTLVL), H (VMAPRILIL), I (VMAPRSLIL), J (VMAPRSLLL), K (VMAPQSLLL), L (VMAPRTLFV) and M (VMAPRTLVL) were synthesized by GLS Biochem (China) and purified to > 90% by HPLC. Peptides were dissolved in DMSO and stored at − 80 °C. UV-sensitive peptides were synthesized with the modified J amino acid, 3-amino-3-(2-nitro)phenyl-propionic acid also purchased from GLS Biochem.

### Production of biotinylated HLA-E/peptide monomers by batch protocol

The soluble HLA-E/peptide complexes were produced as previously described^23,24^. Briefly, recombinant HLA-E*01:01 heavy chain and ß2microglobulin light chain were produced as inclusion bodies in bacterial strain BL21(DE3) and TG1, respectively, resuspended in 8 M urea buffer and stored at − 80 °C. The heavy and light chains were then refolded in vitro with the peptide of interest during 5 days at 4 °C with slow agitation. The solution was concentrated on a 10 kDa Amicon membrane (Millipore, Bedford, MA) and dialyzed overnight at 4 °C against 10 mM Tris pH 8 buffer. The monomers were then biotinylated with BirA enzyme (Avidity, Denvers CO) for 5 h at 30 °C and desalted on HiPrep 26/10 column (Cytiva) with 10 mM Tris–HCl pH 8, 150 mM NaCl buffer. Purification was completed with a size exclusion step on a Superdex 200 10/300 GL column (Cytiva). Biotinylation of the HLA-E/peptide complex was assessed by tetramerization assay with streptavidin (Sigma Aldrich) after one hour incubation at room temperature and injected onto the Superdex 200 10/300 GL column. Purified complexes were stored at − 80 °C until use.

### Peptide exchange

The UV-Induced MHC peptide exchange protocol was adapted from a method previously described by Rodenko^31^ by changing the exchange buffer with 10 mM Tris–HCl pH 8 and 150 mM NaCl. A pre-humidification of the UV chamber (Crosslinker Uvitec) was performed by running it for one hour with a beaker of distilled water to limit samples evaporation. For each experiment, two controls were done, one by exposure of HLA-E/pUV monomers without rescue peptide and the other one with HLA-E/pUV monomers mixed with a positive Melan-A control peptide (ELAGIGILTV, Eurogentec). Each test peptide was mixed in large excess (100X) with HLA-E/pUV monomers in polypropylene V-shaped 96-well plates (Greiner Bio one, 651201) in a final reaction of 25 to 200 µL. To avoid evaporation, the wells surrounding the test wells were filled with distilled water. The UV exchange samples were then incubated for 1 h on UV chamber at 365 nm. After UV exposure, the samples were centrifugated 5 min at 3300 g to remove any aggregates. Samples were collected, and the volume was accurately measured to correct the concentration if evaporation occurred. Samples were stored at 4 °C.

### HLA-E/peptide complexes quantification by ELISA

Exchange efficiency was monitored by ELISA as previously described^31^. Briefly, Maxisorp immunoplates were coated with 2 µg/mL streptavidin at 4 °C overnight and then saturated with PBS containing 0.5% BSA and 0.05% Tween 20 for 2 h at room temperature. A standard titration curve was obtained with biotinylated HLA-E/Peptide D monomer produced according to a batch protocol for concentrations ranging from 6.25 to 400 ng/mL (duplicates). The exchange reaction was diluted to a theoretical concentration of 41 ng/mL. After pre-incubation of the standard controls and samples with saturation buffer for 30 min at room temperature, 50 µL per well were dropped off in duplicate and incubated for 2 h at room temperature. After 3 washes with PBS containing 0.05% Tween 20, the test was revealed with 50 µL of saturation buffer pre-incubated with horseradish peroxidase-conjugated anti-ß2m monoclonal antibody (BioRad, 6240-0354P) at 1 µg/mL and incubated for 2 h at room temperature. The enzyme substrate (Hydrogen peroxide, R&D) was added, the reaction was stopped by addition of H_2_SO_4_ 2 M and the plate was read at 450 nm. The concentration of each sample was calculated from the calibration curve after subtraction of the negative control without rescue peptide and after correction for volume evaporation. The exchange rate was then obtained by comparison with the theoretical concentration of 41 ng/mL.

### HLA-E/peptide complexes quantification by SEC

Quantification of the concentration of peptide/HLA-E complexes after UV exchange was also performed using an Alliance HPLC system 2695e (Waters Corporation) with a PDA detection system. Samples were injected onto an X-Bridge Protein BEH 200 Å, 3.5 µm, 7.8 mm*300 mm size exclusion column (Waters Corporation). A calibration curve was generated by injecting eight amounts of HLA-E/Peptide D complexes and the calibration curve was obtained with Empower 3 software (Waters Corporation). Each HLA-E monomer produced by UV-induced peptide exchange was injected and automatically quantified by the HPLC software from this reference curve.

### Tetramer staining of HCMV-specific HLA-E-restricted T cells

HLA-E_UL40_-restricted T cells were magnetic-sorted from PBMCs of a kidney transplant patient as previously described^14^. HLA-E monomers complexed with the following 6 peptides, VMAPRTLIL, VMAPRTLLL, VMAPRTLVL, VMAPRTVLL, VMAPRSLIL and VMAPRSLLL, were tetramerized with APC-labeled streptavidin (BD Biosciences, Le Pont de Claix, France). T cells were pre-incubated with a blocking anti-CD94 antibody (clone HP-3D9, 5 µg/mL) for 20 min at 4 °C. T cells were then incubated with the different APC-labelled HLA-E-tetramers (10 µg/mL, 30 min, 4 °C), before being stained (30 min, 4 °C) with anti-CD8α (clone RPA-T8, FITC, 0.1 µg/mL, BioLegend). Acquisition was performed using on a BD LSR II and analyses were performed with BD FACSDiva software 8.0.2. Viable cells were first gated based on their morphology in FSC-A/SSC-A. Doublets were then excluded using FSC-A/FSC-H and SSC-A/SSC-H dot plots before analysis. Results are expressed as percentages of CD8^+^Tetramer^+^ cells and median fluorescence intensity (MFI).

### Mass spectrometry analysis

Analyses were carried out on a Synapt™ G2 HRMS Q-TOF mass spectrometer equipped with an electrospray interface (ESI) and an Acquity H-Class® UPLC™ device (Waters Corporation). Samples (10 µL, ~ 100 µg/mL) were injected onto a BEH C18 column (1.7 µm; 2.1 × 150 mm, Waters Corporation) maintained at 60 °C. Compounds were separated with a linear gradient of mobile phase B (100% acetonitrile, 0.1% formic acid) into mobile phase A (5% acetonitrile, 0.1% formic acid) at a flow rate of 250 µL/min. Mobile phase B was kept constant for 1 min at 5%, increased linearly from 5 to 95% for 16 min, kept constant for 1 min, returned to the initial condition over 1 min, and kept constant for 1 min before the next injection. The full-HRMS mode was applied for peptide/protein detection (mass-to-charge ratio (*m/z*) range 50–1200) at a mass resolution of 25,000 full-widths at half maximum. Ionization settings were as follows: positive ionization; capillary voltage, + 3 kV; cone voltage, 30 V; desolvation gas (N_2_) flow rate, 500 L/h; desolvation gas/source temperatures, 550 °C and 20 °C. A solution of leucine enkephalin (2 µg/mL, 50% acetonitrile) was infused at a constant flow rate of 10 µL/min in the lockspray channel, allowing correction of the measured *m/z* throughout the batch (theoretical *m/z* 556.2771). Data acquisition and processing were achieved using MassLynx software (version 4.1, Waters Corporation). Protein molecular weights were estimated by deconvolution (MaxEnt1 extension software). Peptide sequences were determined or confirmed by tandem mass spectrometry (MS/MS). For MS/MS analyses, samples were reinjected and precursor ions were selected for fragmentation (collision set at 30 eV).

## Supplementary Information

Supplementary Figure S1.

Supplementary Figure S2.

Supplementary Legends.
