# Supplementary material for: A novel and efficient approach to high-throughput production of HLA-E/peptide monomer for T-cell epitope screening
Source: Sci Rep. 2021 Aug 26;11:17234. doi: 10.1038/s41598-021-96560-9 (PMC8390762; doi:10.1038/s41598-021-96560-9)
Supplement: Supplementary file 1 — Supplementary Figure S1. [file 41598_2021_96560_MOESM1_ESM.pdf]

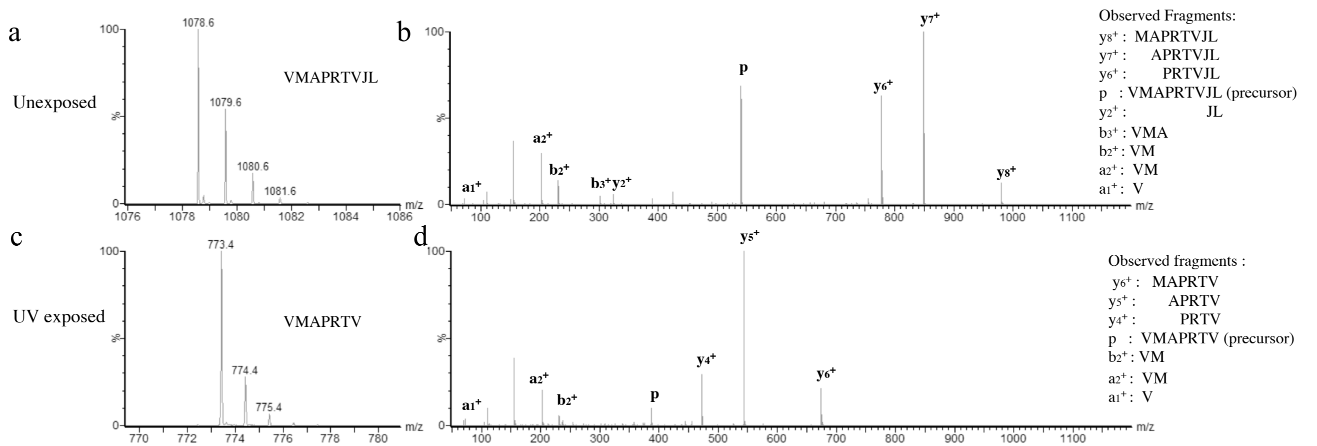
a
Unexposed
VMAPRTVJL
1078.6
1079.6
1080.6
1081.6
m/z
b
Observed Fragments:
y8+ : MAPRTVJL
y7+ : APRTVJL
y6+ : PRTVJL
p : VMAPRTVJL (precursor)
y2+ : JL
b3+ : VMA
b2+ : VM
a2+ : VM
a1+ : V
c
UV exposed
VMAPRTV
773.4
774.4
775.4
m/z
d
Observed fragments :
y6+ : MAPRTV
y5+ : APRTV
y4+ : PRTV
p : VMAPRTV (precursor)
b2+ : VM
a2+ : VM
a1+ : V
