# Supplementary figures and images for: A novel and efficient approach to high-throughput production of HLA-E/peptide monomer for T-cell epitope screening
Source: Sci Rep. 2021 Aug 26;11:17234. doi: 10.1038/s41598-021-96560-9 (PMC8390762; doi:10.1038/s41598-021-96560-9)

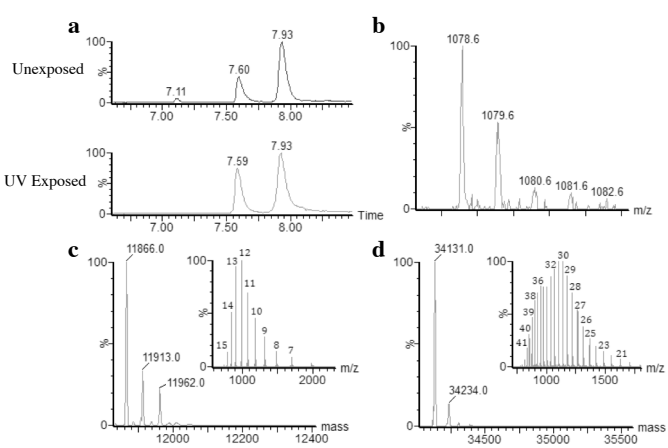

Supplement: Supplementary file 2 — Supplementary Figure S2. [file 41598_2021_96560_MOESM2_ESM.pdf]
