# Supplementary material for: A novel and efficient approach to high-throughput production of HLA-E/peptide monomer for T-cell epitope screening
Source: Sci Rep. 2021 Aug 26;11:17234. doi: 10.1038/s41598-021-96560-9 (PMC8390762; doi:10.1038/s41598-021-96560-9)
Supplement: Supplementary file 3 — Supplementary Legends. [file 41598_2021_96560_MOESM3_ESM.docx]

**Supplementary Figure Legends**

**Supplementary S1 : Characterization of cleavage pUV peptide D2 by LC-MS/MS**

Peptides were diluted to a concentration of 64.8 µM and injected onto a C18 column. Mass spectrometry profiles are shown for native pUV D2 peptide (**a, b**) and the cleaved D2 peptide (**c, d**). a and c represent the LC profiles with the peptide sequence. b and d show the MS/MS profiles with the identified fragments allowing sequence deduction.

**Supplementary S2 : Characterization of HLA-E/pUV peptide D2 complexes by mass spectrometry**

The photosensitive monomer HLA-E/pUV peptide D2 was injected onto a C18 column to analyze its components. **a)** LC profiles of the monomer before and after UV exposure. **b**) Mass spectrum analysis of the D2 peptide eluted in the 7.11 min peak of the unexposed monomer LC profile. **c**, **d**) Deconvoluted mass spectra obtained from the analysis of the chromatographic peak at 7.6 min (c) and 7.9 min (d) with mass spectra in insert.
